# Digital health interventions to improve eating behaviour of people with a lower socioeconomic position: a scoping review of behaviour change techniques

**DOI:** 10.1186/s40795-022-00635-3

**Published:** 2022-12-08

**Authors:** Amber Ronteltap, Andrea J. Bukman, Gera E. Nagelhout, Roel C. J. Hermans, Karen Hosper, Annemien Haveman-Nies, Remko Lupker, Catherine A. W. Bolman

**Affiliations:** 1 Knowledge Centre Healthy and Sustainable Living, University of Applied Sciences Utrecht, P.O. box 12011, 3501 AA Utrecht, The Netherlands; 2 IVO Research Institute, The Hague, The Netherlands; 3 Department of Health Promotion, CAPHRI Care and Public Health Research Institute, Maastricht University, Maastricht, The Netherlands; 4 Department of Health Promotion, NUTRIM School of Nutrition and Translational Research in Metabolism, Maastricht University, Maastricht, The Netherlands; 5 Netherlands Nutrition Centre, The Hague, The Netherlands; 6 Pharos, Utrecht, The Netherlands; 7 Consumption and Healthy Lifestyles Group, Wageningen University & Research, Wageningen, The Netherlands; 8 Faculty of Psychology, Open University of the Netherlands, Heerlen, The Netherlands

**Keywords:** eHealth, Intervention, Eating behaviour, Behaviour change technique (BCT), Social class, Scoping review

## Abstract

Specific approaches are needed to reach and support people with a lower socioeconomic position (SEP) to achieve healthier eating behaviours. There is a growing body of evidence suggesting that digital health tools exhibit potential to address these needs because of its specific features that enable application of various behaviour change techniques (BCTs). The aim of this scoping review is to identify the BCTs that are used in diet-related digital interventions targeted at people with a low SEP, and which of these BCTs coincide with improved eating behaviour. The systematic search was performed in 3 databases, using terms related to e/m-health, diet quality and socioeconomic position. A total of 17 full text papers were included. The average number of BCTs per intervention was 6.9 (ranged 3–15). BCTs from the cluster ‘*Goals and planning*’ were applied most often (25x), followed by the clusters ‘*Shaping knowledge*’ (18x) and ‘*Natural consequences*’ (18x). Other frequently applied BCT clusters were ‘*Feedback and monitoring’* (15x) and ‘*Comparison of behaviour*’ (13x). Whereas some BCTs were frequently applied, such as goal setting, others were rarely used, such as social support. Most studies (*n* = 13) observed a positive effect of the intervention on eating behaviour (e.g. having breakfast) in the low SEP group, but this was not clearly associated with the number or type of applied BCTs. In conclusion, more intervention studies focused on people with a low SEP are needed to draw firm conclusions as to which BCTs are effective in improving their diet quality. Also, further research should investigate combinations of BCTs, the intervention design and context, and the use of multicomponent approaches. We encourage intervention developers and researchers to describe interventions more thoroughly, following the systematics of a behaviour change taxonomy, and to select BCTs knowingly.

**Supplementary Information:**

The online version contains supplementary material available at 10.1186/s40795-022-00635-3.

## Introduction

Diet and nutrition are major determinants of population health [1]. Promoting a healthy diet is, therefore, one of the key strategies in the primary prevention of noncommunicable diseases like cardiovascular diseases, diabetes, cancer, and obesity [2]. These diseases are strongly socially patterned, disproportionately affecting individuals with a lower socioeconomic position (SEP) [3]. Also, with regards to diet and nutrition, there is abundant evidence that diet quality follows a socioeconomic gradient, with people with a lower SEP showing unhealthier diets consisting of higher sugar, salt and saturated fat intake, and less vegetables, fruits and nuts [4–6]. The drivers of this SEP gradient in diet quality are multiple, diverse, and dynamic, ranging from physiological factors to aspects of the physical, economic, sociocultural and political environment [cf. [7]. There is, however, general consensus that individuals experiencing socioeconomic disadvantage are an important group whose dietary health could benefit from well-developed interventions that tackle their specific needs and barriers.

Diverse issues including health literacy, family and work commitments, costs, and social influences have been identified as barriers and facilitators to the implementation of dietary interventions among those with lower SEP [8–10]. It appears to be difficult for health professionals to effectively target and engage individuals from this target group in their interventions [11]. Individuals with low SEP are less likely both to perceive the need for diet-related advice, and to participate in these interventions than those with a higher SEP [12–14]. Furthermore, those with lower SEP are more likely to drop out after initial participation in interventions [15, 16]. Finally, there is evidence that socioeconomically disadvantaged individuals may experience poorer behaviour change outcomes than those with higher SEP, potentially leading to further intervention-generated inequalities [17–19]. This may partly be explained by differences between social classes in food and nutrition-related attitudes, beliefs, social norms, and knowledge [4, 7, 20, 21]. Apparently, different approaches are necessary to successfully reach and help disadvantaged individuals and achieve better behaviour change outcomes in diet-related interventions.

Digital innovations, such as e-health and m-health,^1^ have facilitated the development of tailored approaches and reaching large populations against relatively low cost per person [22]. Digital health interventions can be designed to modify people’s attitudes and behaviours, and to increase their belief of being able to change the behaviour [23]. They offer great opportunities to adapt interventions to the needs of disadvantaged people by the presentation of bite-sized information in plain language, accompanied by reading functions, appealing visuals and animations, and speech recognition [24]. These features can be used to apply a wide array of behaviour change techniques (BCTs), such as providing information, facilitating goal setting, increasing social support and prompting barrier identification; techniques that may be particularly helpful for low income groups [4]. Moreover, due to high mobile internet penetration rates [25], many people with lower SEP too are likely to have access to digital health interventions [26], whereas their access to guidance by e.g. dieticians might be limited as a result of financial or geographical constraints. Again, however, it is observed that disadvantaged individuals are less likely to use digital health interventions for health promotion and self-management of dietary behaviour [14, 27, 28]. Moreover, evidence as to which BCTs are a good fit for populations with a low SEP is scarce [17, 18], particularly in the field of digital dietary interventions.

The aim of our scoping review, therefore, is to identify the BCTs that are used in digital dietary interventions specifically targeted at individuals with a low SEP. Our review focuses on the techniques applied in the interventions, using Michie et al.’s taxonomy of behaviour change techniques, which includes 93 BCTs which are categorised in 16 clusters [29]. In our review, we include studies that examined digital health interventions aimed to change eating behaviour of people with a lower SEP, or comparing people from different SEP groups. We aim to answer the following questions:

1) Which BCTs are applied in digital health interventions aimed at eating behaviour of people with a lower socioeconomic position?

2) Which of these BCTs coincide with improved eating behaviour among people with a lower socioeconomic position?

This paper reports a scoping review on literature about digital health interventions aiming to change dietary behaviour of people with a low SEP. A scoping review is suited to map the parameters of a particular research area [30], rather than provide conclusive, quantitative answers to specific questions [31]. Scoping reviews explore how a topic has been studied [32], and can summarise findings from a body of knowledge that is heterogeneous in methods or discipline [33].

## Methods

### Protocol

We used the guidelines for scoping reviews (PRISMA-ScR) developed by Tricco et al. [33] from the original PRISMA (Preferred Reporting Items for Systematic reviews and Meta-Analyses) checklist.

### Eligibility criteria

English language peer-reviewed papers that met the following inclusion criteria were eligible for inclusion in our review: 1) empirical research with the primary aim of changing eating behaviour by applying digital interventions (which includes interventions that combine digital with other elements, and excludes studies that did not report actual behaviour change, and interventions targeting multiple lifestyle behaviours), 2) digital health applications at user side (which excludes digital health applications for professionals only (e.g. digital data management)), and 3) report effects on people with a low SEP. For the third criterion, two variants were eligible: 1) the intervention was specifically targeted at people with a low SEP, or 2) the intervention was targeted at multiple SEP groups, and the impact on the low SEP was reported separately. Low SEP was defined in our study as low income, low education, blue collar work, or from deprived neighbourhoods, but no strict cut-off points were used.

### Selection process, data charting, and analysis

We performed the systematic search in the databases MEDLINE, APA PsycInfo, CINAHL Plus with Full Text & Psychology and Behavioral Sciences Collection. This selection was based on coverage of these databases in terms of research disciplines (including nutrition, psychology, behaviour) and adjacent review papers (e.g. [34]). The final search was performed on April 15^th^, 2020. A quick check was done on July 28^th^, 2021, which did not yield any new records to be included.

The search strategy was developed in multiple iterations. It consisted of three building blocks representing the main elements of the review’s aim (see Table 1). The phrases ‘e-health’ or ‘m-health’ had to appear in the title, and we added all specific applications identified in other digital health review papers (e.g. wireless, mobile health, web-based [34–41]). We operationalised dietary behaviour by combining synonyms for diet with diet constituents known to influence diet quality [39, 42, 43]. These diet-related words had to appear in the title. We built the term for the low SEP target group with indicator characteristics (e.g. income, education), and papers were taken into account if they appeared in title or abstract.

**Table 1:** Final search strategy

Building block	Exact search phrase
Digital health intervention	TI ("wireless" or "electronic health" or ehealth or "e-health" or mhealth or "m-health" or “mobile health” or “digital health” or “interactive health communication*” or telehealth or ICT or “information technolog*” or "communication technolog*" or “health application*” or app or apps or “mobile* application*” or “smartphone* application*” or “web application*” or “computer-based” or “computer based” or online or “web-based” or “web based” or “web access” or "internet-based" or "internet based" or "internet-deliver*" or "internet deliver*" or “technology-enabled” or “technology enabled” or “technology-based” or “technology based” or “mobile technolog*” or “technology-supported” or “technology supported” or “technology-integrated” or “technology integrated” or “interactive technolog*” or telecare or telemedicine or telecommunication* or “video calling” or wearable or wearables or tracker or “monitoring device*” or “digital game*” or “online game*” or “mobile game*” or “video game*” or “text messag*” or SMS or “short message service*” or "multimedia messaging service" or MMS or “text-based” or “text based” or “social media*” or new media* or “participatory media*” or tablet* or ipad* or “e-mail*” or email* or website* or “world wide web” or "mobile phone*" or "mobile device*" or smartphone* or "cell phone*" or "cellular phone*" or “personal digital assistant*” or PDA* or “computer-assisted” or “computer assisted” or “online learning” or “virtual realit*” or blog* or “online social network*” or “virtual*” or “chatbot*” or “software” or “digital assistant*” or “embodied agent*” or “embodied conversational agent*” or “interactive agent*” or “interface agent*” or “artificial agent*” or “computer-tailored” or “computer tailored” or “mobile communication*” or “web communication*”)
Dietary behaviour	AND TI (diet* or nutrition* or eating or “consumption” or "food intake" or “food pattern*” or “food habit*” or intake or foods or vegetable* or fruit* or wholegrain or legume* or nut* or dairy or fish or tea or fat* or oil* or coffee or “red meat” or “processed meat” or “sweetened beverage*” or “juice*” or alcohol* or salt* or snack* or energ* or calor* or sugar* or carbohydrate* or fiber* or protein* or nutrient* or micronutrient* or macronutrient* or vitamin* or calcium or chromium or copper or iron or magnesium or manganese or molybdenum or potassium or sodium or zinc or iodine or selenium or fluoride or phosphorus or chloride)
Low SEP	AND AB (“socio-economic” or “socioeconomic” or sep or ses or poverty or income* or “social class*” or “social status” or unemployed or unemployment or job* or employment* or occupation* or “blue-collar” or “blue collar” or “low education” or “low-educat*” or “education level*” or “years of education” or “years of schooling” or disadvantaged or deprived or underprivileged or “social inequalit*” or “social inequit*” or “social disparit*”)

Each record (title and abstract) found with the final search term was independently screened by two of the three reviewers (AR, AJB, RL). Records were excluded where it could be ascertained from the title and/or abstract that they did not meet the inclusion criteria. In case of disagreement between the two reviewers, the full-text version of the record was discussed in a project meeting between all reviewers.

AR, AJB, and RL developed and calibrated a charting form, based on the Template for Intervention Description and Replication (TIDIeR) checklist [44]. Items in the charting form included descriptors of the paper (e.g. year, author, journal), intervention (e.g. aim, target group, BCT), study execution (sample size, study design), and results (e.g. effect on food intake). The BCT coding process comprised several steps. First, AR, AJB, and RL independently coded BCTs, based on the intervention as described in the publication and, when available, in the published study protocol of the included study. This initial coding step was followed by discussion, after which items of dissensus were sent out for feedback to the research group who designed the BCT taxonomy [29]. A member of this group advised on how to proceed.

### Selection of sources of evidence

**Fig. 1:**
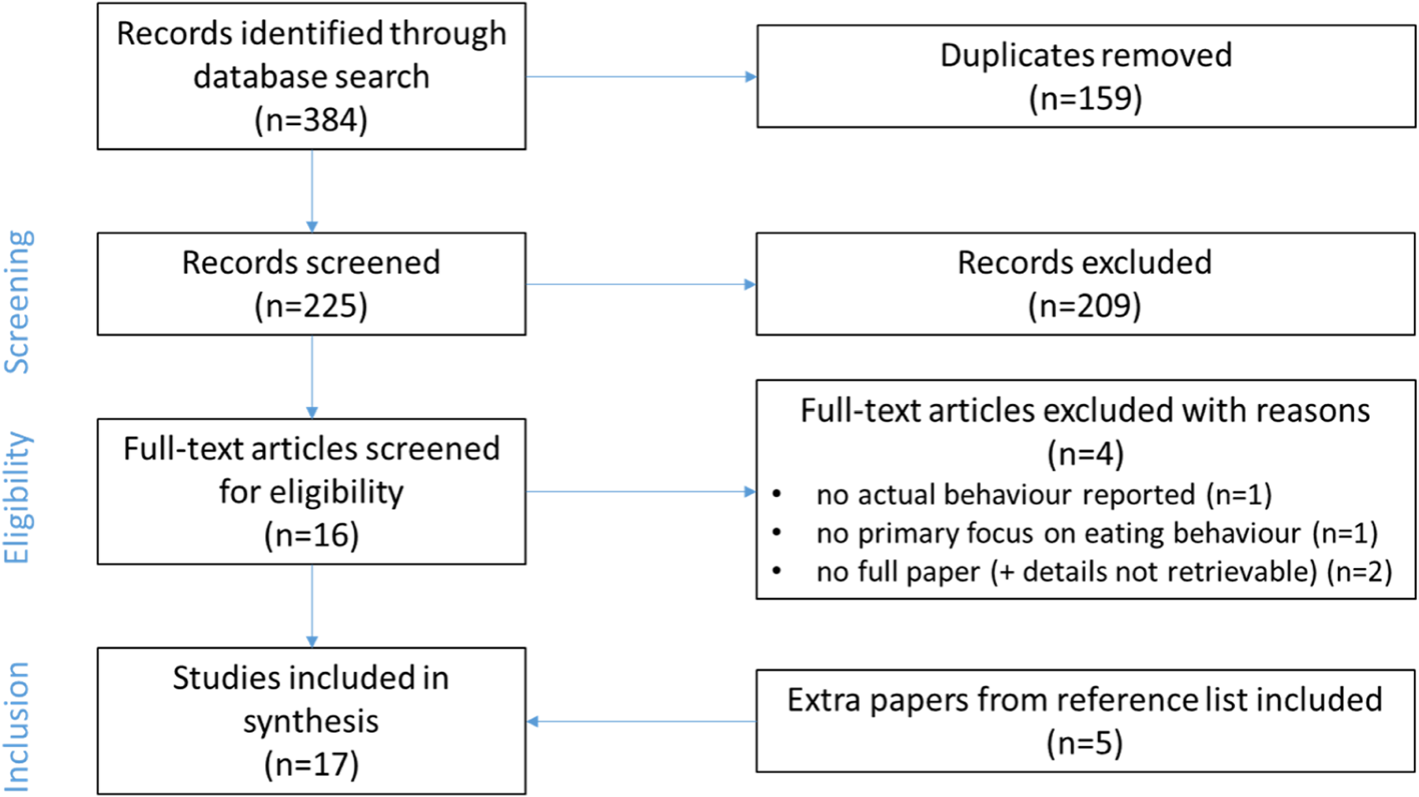
PRISMA flow diagram

The final search resulted in 384 records, of which 159 were duplicates. The remaining 225 records were screened for eligibility using the inclusion criteria, after which the full texts of the remaining 16 articles were retrieved and assessed. A further 4 articles were excluded because they did not fit the inclusion criteria. A manual citation search of the reference lists of the remaining 12 studies was performed, resulting in another 5 articles identified for inclusion. Reasons for missing these articles in our initial search included failure to mention the low SEP target group explicitly in title and abstract, or failure to include comparisons between SEP group in the abstract. As a result, a total of 17^2^ full text papers were eligible for inclusion in this review (see Fig. 1). For 14 of 17 eligible papers, we needed to retrieve secondary papers (mostly study protocols) to identify details of the study.

## Results

### Summary of included studies

Table 2 provides an overview of study characteristics and results of the critical appraisal. Details of all papers can be found in Additional file 1. The final set of papers consists of 17 studies from 6 countries on 3 continents, that together included over 11,000 participants. Results were published over a 17-year period (2004–2020). Most papers were conducted in the USA (*n* = 7), and were published between 2011–2020 (*n* = 15). All papers were based on quantitative data; sample sizes varied considerably (ranging from 26 to 2554 at baseline). The majority of papers (*n* = 12) applied some form of randomised trial (e.g. RCT, block-equivalence, pragmatic). Eleven studies targeted groups with a low SEP specifically, six studies compared multiple SEP groups. Three studies were aimed at adults in general, three at women specifically, five at parents or pregnant women, four at problem drinkers, and two at families as a whole or children. Intake of fruit and vegetables was most often one of the eating behaviours of study (*n* = 11), followed by alcohol (*n* = 5), and unhealthy food (constituent) categories such as snacks, fast food, fat, or sugar (*n* = 4). None of the studies targeted diet as a whole. Ten of the studies measured multiple eating behaviours. Socio-economic position was assessed with income (*n* = 8), education level (*n* = 4), an index of multiple indicators of participants’ area of residence (*n* = 3), or type of housing (*n* = 1). One study, aimed at children, let their subjects self-report their parents’ estimated socio-economic status, which was cross-checked by researchers. As the research designs were heterogeneous, we used the Mixed Methods Appraisal Tool to indicate the quality of the studies [45]. The quality of 13 out of 17 studies was evaluated moderate or high (score of 3 or higher on a 5-point index scale).

**Table 2:** Study characteristics and critical appraisal

**Characteristic**		**Number of papers (*n* = 17)**
Country	USA UK The Netherlands India Japan Malaysia	7 2 5 1 1 1
Publication year	2000–2005 2006–2010 2011–2015 2016–2020	1 1 7 8
Study design	Randomised trial (Quasi)experimental Pre-post test	12 3 2
Target group	Adults/women Mothers (to be)/parents Heavy/binge drinkers Families Children WIC clients^*^	6 5 4 1 1 *4*
Targeted eating behaviour (can be multiple in 1 paper)	Fruit and/or vegetables Alcohol Snacks/fast food/sugar/fat Breakfast Salt Whole grain Folic acid	11 5 4 2 2 1 1
Sample size at baseline	< 50 51–250 251–500 > 500	1 4 2 10
Assessment of SEP	Income Education level Neighbourhood index Housing Self-defined parents’ SEP	8 4 3 1 1
Critical appraisal	Low quality Moderate quality High quality	4 7 6

^*^ WIC is a US Government program for special supplemental nutrition for women, infants and children who are at nutritional risk. It was established in 1974, is available in all 50 states, and is eligible for pregnant, postpartum, and breastfeeding women, infants and children up to age 5 who meet income guidelines, and who are determined to be at nutritional risk (https://www.fns.usda.gov/wic)

### Synthesis of results

#### BCTs

The average number of BCTs used per paper was 6.9 (range 3–15). The frequency of specific BCTs used in interventions in our database is shown in Table 3 (for a complete overview of BCTs applied in each paper in the dataset: see Additional file 1). BCTs from the cluster *‘Goals and planning’* were applied most often (25x), of which the BCT ‘Goal setting (behaviour)’ most frequently (10x). Examples include a nutrition behaviour goal-setting section of a website [46], setting goals related to limiting salt intake [47], and goal setting as one of four successive stages to reduce alcohol consumption in an online self-help programme [48]. Other BCTs from this cluster identified in the database were ‘Problem solving’ (7x) (e.g. suggestions for barriers such as time and cost constraints [49], ‘Action planning’ (6x) (e.g. implementation intentions [23, 50], and ‘Review behaviour goal(s)’ (2x) (e.g. the evaluation of previously formulated behavioural goals [23, 50]. Studies that applied a BCT from this cluster, mostly did this in combination with other BCTs from the same cluster, of which the combination of ‘Goal setting’, ‘Problem solving’, and ‘Action planning’ was applied most frequently (*n* = 5).

**Table 3:** Specification of BCTs used in interventions

	**BCT Cluster**	**Times applied**
1	**Goals and planning** 1.1 Goal setting (behaviour) 1.2 Problem solving 1.4 Action planning 1.5 Review behaviour goal(s)	**25** 10 7 6 2
2	**Feedback and monitoring** 2.2 Feedback on behaviour 2.3 Self-monitoring of behaviour 2.6 Biofeedback 2.7 Feedback on outcome(s) of behaviour	**15** 9 3 1 2
3	**Social support** 3.1 Social support (unspecified)	**5** 5
4	**Shaping knowledge** 4.1 Instruction on how to perform the behaviour 4.2 Information about antecedents	**18** 13 5
5	**Natural consequences** 5.1 Information about health consequences 5.2 Salience of consequences 5.3 Information about social and environmental consequences 5.6 Information about emotional consequences	**18** 13 2 1 2
6	**Comparison of behaviour** 6.1 Demonstration of the behaviour 6.2 Social comparison	**13** 8 5
7	**Associations** 7.1 Prompts/ cues	**1** 1
8	**Repetition and substitution** 8.1 Behavioural practice/ rehearsal 8.2 Behaviour substitution 8.7 Graded tasks	**4** 2 1 1
9	**Comparison of outcomes** 9.2 Pros and cons	**2** 2
10	**Reward and threat**	-
11	**Regulation**	-
12	**Antecedents** 12.1 Restructuring the physical environment 12.2 Restructuring the social environment 12.3 Avoidance/reducing exposure to cues for the behaviour 12.5 Adding objects to the environment	**9** 4 1 2 2
13	**Identity** 13.1 Identification of self as role model 13.2 Framing/ reframing 13.4 Valued self-identity 13.5 Identity associated with changed behaviour	**6** 3 1 1 1
14	**Scheduled consequences**	-
15	**Self-belief** 15.2 Mental rehearsal of successful behaviour	**1** 1
16	**Covert learning**	-

BCTs from the clusters *‘Shaping knowledge’* (18x) and *‘Natural consequences’* (18x), both related to knowledge transfer, were also applied often. We identified ‘Instruction on how to perform the behaviour’ 13x, e.g. in Delrahim-Howlett et al. [51] (specific tips on sensible drinking), and in Neuenschwander et al. [46] (recipe video demonstrations). ‘Information about antecedents’ (5x) was applied e.g. in the intervention reported by Power & Bersamin [52], in which fruit and vegetable discounts at grocery stores were announced. Most common BCT from the cluster *‘Natural consequences’* was ‘Information about health consequences’ (13x), which was applied by e.g. Au et al. [47] by providing participants information about why skipping breakfast can lead to poorer health. Other BCTs from this cluster we identified in our database were ‘Salience of consequences’ (2x) [53, 54], ‘Information about social and environmental consequences’ (1x) [51], and ‘Information about emotional consequences’ (2x) [23, 50].

BCTs from the cluster *‘Feedback and monitoring’* were applied 15x. ‘Feedback on behaviour’ (9x), for example, was used by Gootjes et al. [55], by showing results from screening sessions compared to previous screening sessions on participants’ personal page. ‘Self-monitoring’ was applied 3x (in e.g. Nakamura, Inayama, Harada, & Arao [56]), ‘Biofeedback’ 1x (blood test reports in Kaur et al. [57]), and ‘Feedback on outcome(s) of behaviour’ 2x (in e.g. Delrahim-Howlett et al. [51]), where participants were presented with the financial cost associated with the number of alcohol units they consumed in the previous 2 weeks).

The BCT cluster *‘Comparison of behaviour’* was used 13x, and consisted of ‘Demonstration of the behaviour’ (8x) (e.g. in Au et al. [47]), where they showed participants how to use certain foods to make healthy breakfast) and ‘Social comparison’(5x) (e.g. in Crombie et al. [53]), where normative information was provided about others’ behaviours and experiences).

Other BCT clusters were applied less than 10 × in the included papers. BCTs from *‘Antecedents’* present in our dataset were ‘Restructuring the physical (4x) or social (1x) environment’, ‘Reducing exposure to cues’ (2x), and ‘Adding objects to the environment’ (2x). Examples include providing measuring spoons to participants, recommending to place fruits and vegetables on the table, and to keep snacks less visible [57]. BCTs from the cluster *‘Identity’* were ‘Identification of self as role model (3x), ‘Framing/ reframing’ (1x), ‘Valued self-identity’ (1x), and ‘Identity associated with changed behaviour’ (1x). Examples include motivating parents to be positive role models for their children by consuming fruits and vegetables, and portraying shopping and cooking with children as a great way to spend quality time together [52]. The BCT from ‘*Social support’* was ‘Social support (unspecified)’ (5x), for example a moderated peer-to-peer discussion forum [48]. BCTs from ‘*Repetition and substitution’* were ‘Behavioural practice/ rehearsal’ (2x), ‘Behaviour substitution’ (1x), and ‘Graded tasks’ (1x). Examples include downloadable recipes after recipe video demonstrations [46], or setting small tasks towards a goal [56]. The BCT from the cluster ‘*Comparison of outcomes’* was ‘Pros and cons’ (2x) (e.g. letting participants choose two behaviour-specific advantages and disadvantages that are most important to them from a predefined list [23, 50]. The BCT from *‘Associations’* was ‘Prompts/cues’ (1x) (providing a pictorial calendar for display in the kitchen to remind participants of using less fat, sugar and salt while cooking). The BCT from *‘Self-belief’* was ‘Mental rehearsal of successful behaviour’ (1x) (imagining the planned increase in vegetable intake [56].

Four BCT clusters were not applied at all, namely ‘*Reward and Threat’* (e.g. material incentive), *‘Regulation’* (e.g. reduce negative emotions), *‘Scheduled consequences’* (e.g. behaviour cost), and *‘Covert learning’* (e.g. imaginary punishment).

#### Effects on eating behaviour

All included studies were based on quantitative data, however, direct comparison of the effectiveness of reported interventions is complicated by the highly diverse interventions, target groups and research designs. For example, the duration of interventions varied between a one-off short session [47] and a 12 months period [48]. The timing of effect measurements was just as diverse (ranging from directly after the intervention [52] to 12 months afterwards [53]). The comparison group varied as well (if present at all); whereas some studies compared a digital health intervention with a control group (e.g. Shukri, Zin, Zainol, Said, & Rajali [58]), other studies compared the digital health intervention with another type of treatment (e.g. in-person [47]) or another type of digital health intervention (personalised vs. generic) (e.g. Delrahim-Howlett et al. [51]). We focus the results section on whether the digital health intervention was effective in changing dietary behaviour of the low SEP target group.

Of the 17 included papers, four papers found no change [52] or no effect in the low SEP group compared to a control group [23, 53, 54]. The other 13 studies observed at least some positive effects of the digital health intervention on eating behaviour among persons with a low SEP. Seven studies showed mixed results, either because the positive changes were not observed for all included dietary behaviours [46, 47, 49, 50, 59], or because the established effects were not sustained over time [56, 58].

Six studies reported a consistent positive effect of the digital health intervention on the target group’s eating behaviour. Kaur et al. [57] showed that their multi-channel intervention was effective in improving intake of fat, sugar, salt, fruit, and vegetables among all included socioeconomic groups. Gootjes et al. [55] concluded that overall, the proportion of women with inadequate food intakes decreased as a result of their mobile health coaching program, and, specifically, that women with a lower SEP were more likely to improve inadequate vegetable intake than women with a higher SEP. In Fielden’s [60] experiment using online recommendations, self-affirmed mothers with a low SEP reported higher fruit and vegetable intake than non-self-affirmed low SEP mothers. Risky-drinking low-income women participating in Delrahim-Howlett’s [51] web-based intervention significantly reduced their alcohol consumption, in both the generic and the personalised digital health intervention treatment group. Participants in the online treatment group of Au’s [47] trial showed a greater increase in their frequency of eating breakfast than participants in the in-person treatment group. The web-based self-help intervention reported by Riper et al. [48] resulted in a greater reduction in alcohol intake of problem drinkers from all SEP groups than the control group who received an online brochure. Although an effect was found in the low SEP group, they found an inversed education effect; highly educated users were slightly more likely to benefit from the intervention.

Table 4 shows the characteristics of the included interventions in terms of average number of BCTs and BCT clusters applied. Visual inspection suggests that interventions that were effective in changing dietary behaviour of our target group, used less BCTs than both ineffective interventions and interventions that showed some effects. However, a Kruskal–Wallis test showed no significant differences in mean number of BCTs used between the three groups (H = 3.86, *p* = 0.145).

**Table 4:** Average number of BCTs per intervention and times a cluster of BCTs was applied, per category of intervention effectiveness and in total

Effects?	N	Avg#BCTs^*^	1. Goals & planning^**^	2. Feedback & monitoring	3. Social support	4. Shaping knowledge	5. Natural consequences	6. Comparison of behaviour	7. Associations	8. Repetition and substitution	9. Comparison of outcomes	12. Antecedents	13. Identity	15. Self-belief
No	4	9,5	3	2	0	2	4	3	0	0	1	2	2	0
Some^***^	7	7,1	5	5	2	6	5	5	0	3	1	1	2	1
Yes	6	4,8	2	3	3	5	4	2	1	1	0	2	1	0
Total	17	6,9	10	10	5	13	13	10	1	4	2	5	5	1

^*^ *Avg#BCTs* Average number of BCTs applied per intervention

^**^Numbers in column headings refer to Michie’s BCT taxonomy clusters

^***^Studies were classified as ‘some effects’ when positive changes were not observed for all included dietary behaviours, or when established effects were not sustained over time

BCT cluster 1 ‘*Goals and planning*’, a cluster from which BCTs were applied most frequently overall (25 times, see Table 3), was used in only one third of effective interventions, and in three quarters of ineffective interventions. The same pattern can be seen for Cluster 6 ‘*Comparison of behaviour*’. Conversely, BCTs from Cluster 3 ‘*Social support*’ were applied in half of the effective interventions, in only 2 out of 7 partly effective interventions, and in none of the ineffective interventions. BCTs from Clusters 4 ‘*Shaping knowledge*’ and 5 ‘*Natural consequences*’ were used relatively often in all three categories of included interventions. Both the small number of studies and the skewed distribution of BCTs over the included studies impeded statistical testing of associations between BCT cluster and intervention effectiveness.

## Discussion

### Summary of results

This scoping review aimed to identify potentially successful BCTs applied in digital dietary interventions targeted at individuals with a lower socioeconomic position. In the 17 identified papers, on average 7 BCTs were applied. None of the interventions used more than 15 or less than 3. Relatively few BCT clusters were used very often. The BCT cluster ‘*Goals and planning’* was applied most often with ‘Goal setting’ as most frequently applied technique, followed by ‘Problem solving’ and ‘Action planning’. Second in frequency were the BCT clusters *‘Shaping knowledge’* and *‘Natural consequences’*, both related to the provision of information, most frequently about the health consequences of the dietary behaviour. The clusters ‘*Feedback and monitoring*’ and ‘*Comparison of behaviour*’ were applied third most often. Other clusters were applied in less than 10 interventions, or not at all.

Thirteen out of 17 included papers reported positive effects on dietary intake behaviour. Yet, effects were not consistent in most studies, i.e. only temporary or partial. Six out of thirteen papers reported consistent effects for the duration of the study: two on alcohol consumption, one on fruit and vegetable consumption, two on multiple eating behaviours, and one on breakfast eating. There were only indicative associations between the effectiveness of interventions and the identified BCTs. BCTs related to goals and planning (cluster 1), and comparison of behaviour (cluster 6) appeared relatively more often in ineffective than in effective interventions, and BCTs related to social support (cluster 3) appeared more often in effective than in ineffective interventions. However, the small number of studies impede any conclusions considering effectiveness of BCTs. This implies that there is no conclusive evidence for the successfulness of certain BCTs applied in dietary digital health interventions targeted at individuals with a low SEP.

### Interpretation

The heterogeneity in effects was comparable to the findings of reviews of digital dietary interventions in the general population [39, 61–63]. Rodriguez et al. [63] revealed larger effect sizes in digital health interventions with seven or more BCTs compared to interventions with fewer BCTs. However, the effective interventions in our review seemed to have applied less BCTs than ineffective interventions, although this difference was not statistically significant. Given that this latter indication is in line with Michie et al. [18], who also suggested that for low-income groups, using few BCTs might be more effective for changing behaviour, the question as to the optimal number of BCTs for low SEP target groups remains open for scientific investigation.

Other reviews have sought indications for effective BCTs in low SEP target groups, although not in the context of digital health interventions. Shagiwal and colleagues [64] found five self-regulatory BCTs to be effective in interventions for soft drink intake by disadvantaged adolescents in their meta-analysis: feedback, goal-setting, action planning, self-monitoring and problem-solving/barrier identification. In the meta-analysis of Bull et al. [17], the BCTs self-monitoring, delivery through personal contact, and targeting multiple dietary sub-behaviours were associated with increased effectiveness of healthy eating interventions. Providing feedback, information about emotional consequences or using prompts and cues were associated with reduced effectiveness. Again, these results are mixed (e.g. regarding feedback), and not in line with the suggested associations in our study, for example regarding ‘*Goals and planning*’ BCTs (see Table 4). Michie et al. [18] reviewed the effectiveness of BCTs in low-income groups for health behaviour change in general. Their results also did not give clear indications which BCTs are effective for low income group, nor did they include digital health interventions. Other types of publications made suggestions too considering how to help people with a low SEP to change their eating behaviour [4, 65]. Action plans, the provision of social and emotional support, and stress reduction have been mentioned as promising strategies, though again, not in the context of digital health interventions, and only on theoretical grounds.

In a meta-analysis regarding health behaviour change in a general population, Dusseldorp et al. [66] identified particular combinations of BCTs that determine success in changing behaviour. Most effective combinations were: 1) ‘Provide information about link between behaviour and health’ with ‘Prompt intention formation’, and 2) ‘Provide information about link between behaviour and health’ with ‘Provide information on consequences’ and ‘Use of follow-up prompts’. Least effective interventions were those providing feedback on performance without further instruction and guidance. Unfortunately, we were not able to assess the effects of the combinations of BCTs in our review, due to the small number of studies eligible for inclusion, and the large variation in BCTs. Whether the specific BCT combinations reported by Dusseldorp are as effective for low SEP groups, needs further study.

Our results, together with those of other studies that attempted to unravel the effectiveness of certain BCTs in changing health behaviours, draw an inconclusive picture. In our study, the small number of interventions and the heterogeneity of the interventions with regard to target groups, dietary behaviours, and interventions, have complicated firm conclusions. Some interventions only included digital components whilst others combined digital health interventions with face-to-face elements or written materials. Similar BCTs were used in both effective and ineffective interventions, which might have masked possible effects. Also, other studies reach variable conclusions, for example regarding the effectiveness of providing feedback. This implies that it is not only the BCT itself that preconditions effect, so does the context in which the BCT is delivered, in which combinations, and how it is translated into a practical strategy [67].

The categorisation process of BCTs was often difficult, because this element had received little attention in intervention descriptions. Many descriptions are brief and lack information on active BCT components of the interventions [67–69]. Another complicating factor is that our review concerns digital health interventions, in which the design features are also a precondition for effectiveness [70], especially for those with low (e-)health literacy [71], which is more often the case in low SEP groups [27, 72, 73]. Furthermore, adherence to intervention elements has not been taken into account. For future reviews it is recommended to also include the parameters of adherence and digital health intervention design [70]. It would be important to examine which (combination) of BCTs and practical strategies enhance adherence and engagement among the target group as effectiveness studies often suffer from type 3 error. Potential parameters to measure adherence include the number of logins, the number of different days participants used the technology, the time spent on the technology, the number of modules started or completed, or the number of different elements that were accessed or used [74]. These data should be interpreted with care, as the amount of use that is needed to obtain the desired outcomes may vary across different user groups [75].

## Strengths and limitations of the scoping review

An important strength of the present scoping review is that most studies used an RCT to assess the intervention effects, which serves as the golden standard for effect studies. Another strength is the use of the renowned BCT taxonomy of Michie et al. to lay bare the BCTs that are used in dietary interventions for people with a low SEP. This target group has been underrepresented in reviews on digital health interventions in general and on dietary behaviours specifically, although their eating behaviour is least healthy in comparison with other groups [4–6], and digital health interventions provide great opportunities to be customised to the needs of this group [24]. Therefore, the unique combination of BCT assessment in digital health interventions for people with a low SEP’s eating behaviour, with the inclusion criterion of having measured actual behaviour change, is an asset of this review. Lastly, although 7 of 17 studies were conducted in the USA, our review includes papers from three continents (North-America, Europe, Asia), which enhances generalisability of results.

Our scoping review is also prone to limitations. First, the diversity in the studies concerning target group, dietary behaviours, and the timing of effect measurements have seriously complicated the comparison of BCTs and intervention effects. Also, the fact that about a third of the interventions combined digital with face-to-face elements has impeded the comparison. Consequently, it was difficult to unveil what distinguishes effective from non-effective BCTs. A second limitation concerns the inventory of BCTs that were used. One aspect in this regard is that tailoring has not been included in Michie’s taxonomy, while it was often reported as a behaviour change technique in interventions in the studies included in our review. Moreover, tailoring is considered an important technique in other behavioural change taxonomies (e.g., in the Intervention Mapping approach [67, 76]). It might therefore improve the usability and comparability of Michie’s BCT taxonomy to also include tailoring.

Another aspect is that the coding process might have been influenced by different interpretations of the names and definitions provided by the papers’ authors for the intervention elements. This might have occurred despite the standardised coding method in Michie’s taxonomy, our rating procedure with two independent raters, and the fact that Michie and the intervention developers were consulted in case of ambiguity. It might have induced bias toward common BCTs as these are recognised more easily than rare BCTs. Third, the studies that were included not only targeted low SEP groups; 6 of 17 studies compared multiple SEP groups, which might have affected the power of our analyses negatively.

## Conclusions

This scoping review provided insight in BCTs used in digital dietary interventions for low SEP target groups, and investigated indications of the effectiveness of those BCTs. The review delivers an overview of how many and which BCTs were applied, but evidence for the success of specific BCTs could not be laid bare. More specific studies are required that focus on the needs and contexts of people with a low SEP. In addition to the assessment of the effectiveness of separate BCTs, it is recommended to investigate combinations of BCTs, the intervention design and context, and the use of multicomponent approaches. To unfold the effects of the interventions as well as those of the incorporated BCTs, reach and adherence to the intervention should be considered too.

A take home message for intervention developers, researchers, and practice professionals is to choose BCTs knowingly, and not apply common techniques such as goal-setting or knowledge transfer by default. Social support, for example, could add to intervention effectiveness, but is not often used. Also, intervention developers should not be tempted to apply as many BCTs as possible, since it is unclear whether more BCTs is always associated with more effective interventions. Additionally, in designing digital health interventions, not only BCT selection should be considered carefully, but also design features like graphics, appearance, and layout, and context-specific factors like characteristics of the target group and their environment. Intervention developers and researchers are encouraged to describe the interventions they have developed more thorough, following the systematics of a behaviour change taxonomy (e.g. Kok et al. [67]; Michie et al. [29]), and including design features [70]. Complete intervention descriptions are necessary for revealing what works in digital health interventions aimed at changing eating behaviour of people with a low SEP.

To conclude, this review adds to the literature by providing a first, novel, and specific overview of studies on the potential successfulness of digital health interventions and incorporated BCTs in changing eating behaviour of people with a low SEP. It provided indications for intervention effectiveness and showed which BCTs are frequently applied, such as goal setting, planning, and information provision, and which BCTs are rarely applied, such as social support. Furthermore, the review provided suggestions for topics that need further research. Such research is needed to exploit the potential added value of digital elements in interventions to support healthy behaviours of people with a low SEP. Our work shows that this disadvantaged target group has been understudied in this particular field of research, while they should be prioritised given the magnitude of health disparities.

## Supplementary Information

**Additional file 1.**

## Data Availability

The dataset generated and/or analysed during the current study is available as additional file.

## Footnotes

1 For reasons of conciseness, we use the term *digital health interventions* to indicate both *e-health* and *m-health* from this point onwards, following the WHO definition: digital health is defined as the use of digital, mobile and wireless technologies to support the achievement of health objectives. Digital health describes the general use of information and communications technologies (ICT) for health and is inclusive of both mHealth and eHealth [77].

2 Two papers of Springvloet report the effects of the same intervention, measured at different time points (1 month [50] and 9 months [23] post-intervention). As these are separate publications with different outcomes, we included both in our review.
